# Reinforcement magnitudes modulate subthalamic beta band activity in patients with Parkinson’s disease

**DOI:** 10.1038/s41598-018-26887-3

**Published:** 2018-06-05

**Authors:** Henning Schroll, Andreas Horn, Joachim Runge, Axel Lipp, Gerd-Helge Schneider, Joachim K. Krauss, Fred H. Hamker, Andrea A. Kühn

**Affiliations:** 1 Neurology, Charité - Universitätsmedizin Berlin, Berlin, Germany; 2 Computer Science, Chemnitz University of Technology, Chemnitz, Germany; 3 Psychology, Humboldt University Berlin, Berlin, Germany; 4 Neurosurgery, Medical University Hanover, Hanover, Germany; 5 Neurosurgery, Charité – Universitätsmedizin Berlin, Berlin, Germany

## Abstract

We set out to investigate whether beta oscillations in the human basal ganglia are modulated during reinforcement learning. Based on previous research, we assumed that beta activity might either reflect the magnitudes of individuals’ received reinforcements (reinforcement hypothesis), their reinforcement prediction errors (dopamine hypothesis) or their tendencies to repeat versus adapt responses based upon reinforcements (status-quo hypothesis). We tested these hypotheses by recording local field potentials (LFPs) from the subthalamic nuclei of 19 Parkinson’s disease patients engaged in a reinforcement-learning paradigm. We then correlated patients’ reinforcement magnitudes, reinforcement prediction errors and response repetition tendencies with task-related power changes in their LFP oscillations. During feedback presentation, activity in the frequency range of 14 to 27 Hz (beta spectrum) correlated positively with reinforcement magnitudes. During responding, alpha and low beta activity (6 to 18 Hz) was negatively correlated with *previous* reinforcement magnitudes. Reinforcement prediction errors and response repetition tendencies did not correlate significantly with LFP oscillations. These results suggest that alpha and beta oscillations during reinforcement learning reflect patients’ observed reinforcement magnitudes, rather than their reinforcement prediction errors or their tendencies to repeat versus adapt their responses, arguing both against an involvement of phasic dopamine and against applicability of the status-quo theory.

## Introduction

Three well-established lines of research led us to hypothesize that beta oscillations in the subthalamic nucleus are modulated across reinforcement learning. First, dopamine loss in Parkinson’s disease goes along with enhanced oscillatory activity in the beta frequency band (12 to 30 Hz) that can be recorded from the basal ganglia in patients undergoing deep brain stimulation^1–3^. Dopamine replacement therapy, i.e., treatment with levodopa or dopamine agonists, reduces these beta oscillations in Parkinson’s disease patients^1–3^, pointing at an inverse relationship between tonic dopamine levels and beta activity. Whether *phasic* dopamine levels modulate beta activity as well, has not yet been shown. Such phasic dopamine signals encode reinforcement prediction errors, i.e., differences between received and expected reinforcements^4^. If indeed phasic dopamine modulates beta activity, as previously suggested by Jenkinson and Brown^5^, therefore, reinforcement prediction errors during reinforcement learning should do so, too. We here investigated this hypothesis.

Secondly, it has been shown that large reinforcements go along with larger phasic increases in beta activity at frontocentral EEG electrodes than small reinforcements^6^. In subcortical recordings, however, such a reinforcement-related modulation of beta activity has not yet been shown (while other frequency bands have indeed been reported to be modulated in both animals and humans, [e.g.^7–11^]). Given that motor and frontal cortices are heavily connected to the basal ganglia, it appears likely that reinforcement magnitudes during reinforcement learning do modulate beta activity in the basal ganglia, too. We here investigated this hypothesis.

Thirdly, beta oscillations co-vary with motor performance, both in the motor cortex and in the basal ganglia^2,12–15^. Beta activity decreases between approximately one second before to one second after movement onset and rebounds afterwards for several seconds^16^. In Parkinson’s disease, moreover, tonic dopamine loss goes along both with increased beta activity and with the motor-inhibitory symptoms of rigidity and bradykinesia^1–3^. Based on these findings, it has been hypothesized that beta activity signals the motor system’s propensity to maintain (as opposed to adapt) its current state^17^. In reinforcement learning, there is a similar function involved: with each new response choice, subjects have to decide whether they maintain or adapt their response strategies based on previous reinforcements. To the best of our knowledge, it has not been previously shown whether the status quo theory^17^ is applicable to the context of reinforcement learning. If it is, however, it will imply that during reinforcement learning, beta activity increases when subjects maintain their response strategies based on observed reinforcements, but decreases when they adapt these strategies. We here investigated this hypothesis.

Taken together, therefore, we set out to test the three above-described hypotheses that beta activity is modulated by reinforcement prediction errors, by reinforcement magnitudes and/or by response maintenance versus adaptation. Methodically, we recorded intracranial LFPs from the STN in human Parkinson’s disease patients who performed a reinforcement learning paradigm.

## Results

**Figure 1:**
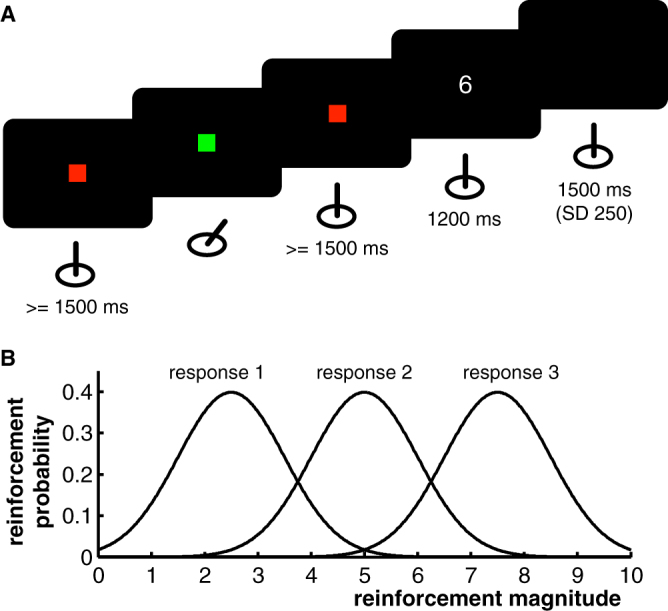
Reinforcement-based learning paradigm. (**A**) Trial setup. At the beginning of each trial, a red fixation square was presented to patients until the joystick had not been moved for at least 1500 ms. This time period served as a baseline in all analyses. The fixation square then turned green, prompting patients to decide for a response and move the joystick accordingly. 500 ms after the decision, the red square was again presented until the joystick had not been moved for 1,500 ms (keep the upcoming period of feedback presentation uncontaminated by post-movement artefacts). Afterwards, the feedback stimulus (number between 0 and 10) was presented for 1,200 ms, followed by an inter-trial interval. (**B**) Feedback probability curves. Each movement direction was mapped onto a Gaussian feedback probability curve that defined the likelihood of different reinforcement magnitudes. Mappings between responses and probability curves remained constant for an average of 20 trials (SD: 3).

Patients performed a reinforcement-based learning paradigm in which they were asked to maximize reinforcements by choosing appropriate responses (Fig. 1A). In each trial of this paradigm, they had to move a joystick to either the left, right or front based on their own decision. Afterwards, a reinforcement stimulus was presented (number between zero and ten). Reinforcement magnitudes were drawn from Gaussian probability curves, where each joystick movement was associated to a particular probability curve. Each 20 trials on average (SD: 3), probability curves were interchanged randomly among directions without prior notice to patients. Curves differed in means, but had equal standard deviations of one (see Fig. 1B).

### Behavioral findings

**Figure 2:**
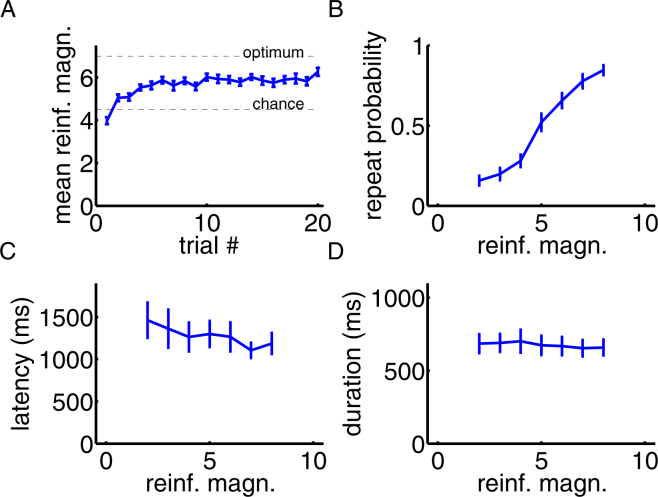
Behavioral results. (**A**) Learning progress. Average reinforcement magnitudes are shown across trials for constant response-reinforcement mappings. Trial #1 corresponds to the first trial after a novel response-reinforcement mapping became valid. (**B**) Response choices are based on reinforcements (Pearson’s r = 0.90, *p* < 0.0001). For different reinforcement magnitudes, the probabilities of repeating the previous trial’s response are shown. Feedback magnitudes below 2 and above 8 are included into the bins of 2 and 8, respectively. (**C**) Response latencies do not significantly depend on reinforcements (r = −0.14, *p* = 0.09). Response latencies are shown for trials following different reinforcement magnitudes. (**D**) Response durations do not significantly depend on reinforcements (r = −0.13, *p* = 0.22). Response durations are shown for trials following different reinforcement magnitudes. Error bars represent SEMs in all sub-plots, computed across patients.

Patients reliably learned our task. Within episodes of constant response-reinforcement mappings, average obtained reinforcements increased in magnitude across trials (Fig. 2A). Moreover, patients clearly based their response strategies on previous reinforcements: reinforcement magnitudes obtained in a given trial correlated significantly with patients’ probabilities of repeating that same response in the following trial (Fig. 2B; Pearson’s r = 0.90, as averaged across patients’ individual correlation values, *p* < 0.001, computed with a non-parametric sign permutation test across patients). Reinforcement magnitudes did not correlate significantly with the following trial’s response latencies (r = −0.14, *p* = 0.09; Fig. 2C) or with response durations (r = −0.13, *p* = 0.22; Fig. 2D).

### Grand-average LFP findings

**Figure 3:**
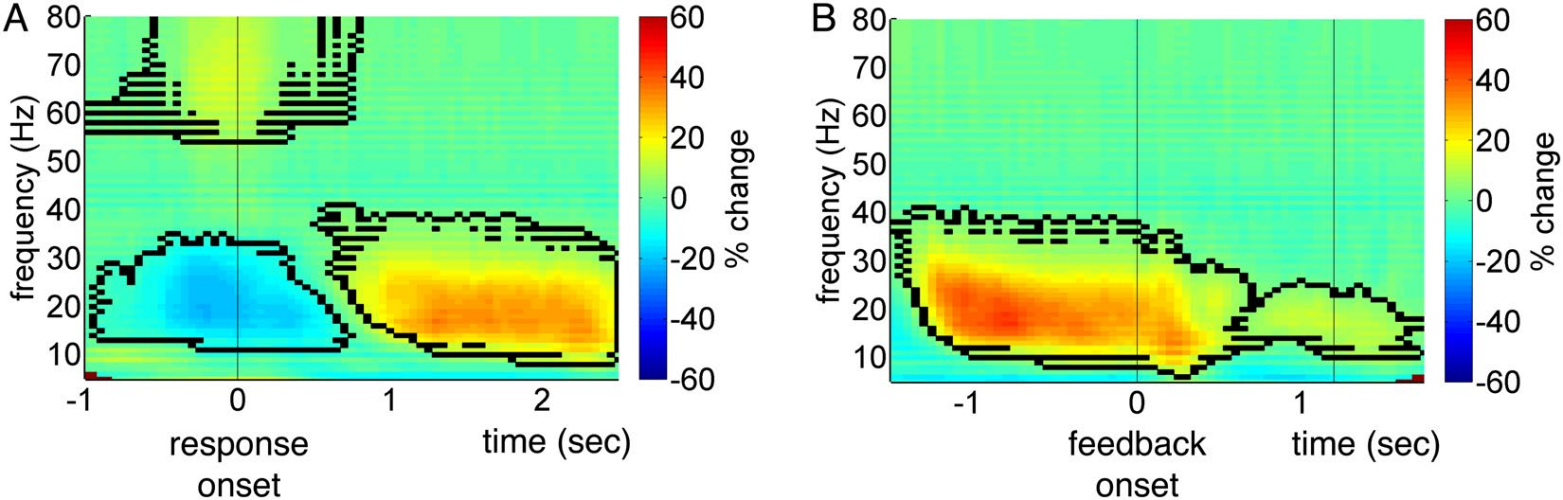
Grand-average time-frequency plots show task-related changes in oscillatory power relative to the baseline interval. Black lines show the borders of significant clusters of power changes as determined with the methods by Maris and Oostenveld^18^. (**A**) Response-locked data. Time point zero denotes response onset. (**B**) Feedback-locked data. Time point zero denotes feedback onset; feedback presentation terminated at 1.2 sec.

Response- and feedback-related changes in oscillatory power, relative to the baseline period, were analyzed using Morlet wavelets. Time-frequency analyses were performed with a time resolution of 50 ms and a frequency resolution of 1 Hz throughout our analyses. Figure 3 plots the grand-average results of these analyses, computed across patients (for corresponding *t*-maps see Supplementary Fig. S1). Our response-locked results are in line with previous findings. We observed a significant movement-related reduction in beta activity during patients’ joystick movements (i.e., between approximately 1000 ms before and 800 ms after response onset; Fig. 3A), *p* = 0.008 (cluster-based statistic, family-wise-error-rate (FWER) corrected)^18^. Moreover, a significant post-movement increase in beta activity starting approximately 800 ms after the movement became apparent, *p* < 0.001. Finally, a significant increase in gamma activity around movement onset was observed, *p* = 0.006. Locking LFP data to response *termination* (instead of response onset) confirmed significance of all three clusters and moreover showed response termination to be approximately in line with the end of the beta decrease and the beginning of the beta increase (Supplementary Fig. S2). In feedback-locked analyses, we again found the prominent post-movement increase in beta activity that stretched until approximately 2000 ms after feedback onset, *p* < 0.001 (Fig. 3B). Upon visual inspection, it appeared to consist of both the actual post-movement beta increase and of another, partly overlapping, but less powerful beta increase during feedback presentation that differed in spectral frequency.

### Correlations of feedback-locked LFPs with behavioral parameters

**Figure 4:**
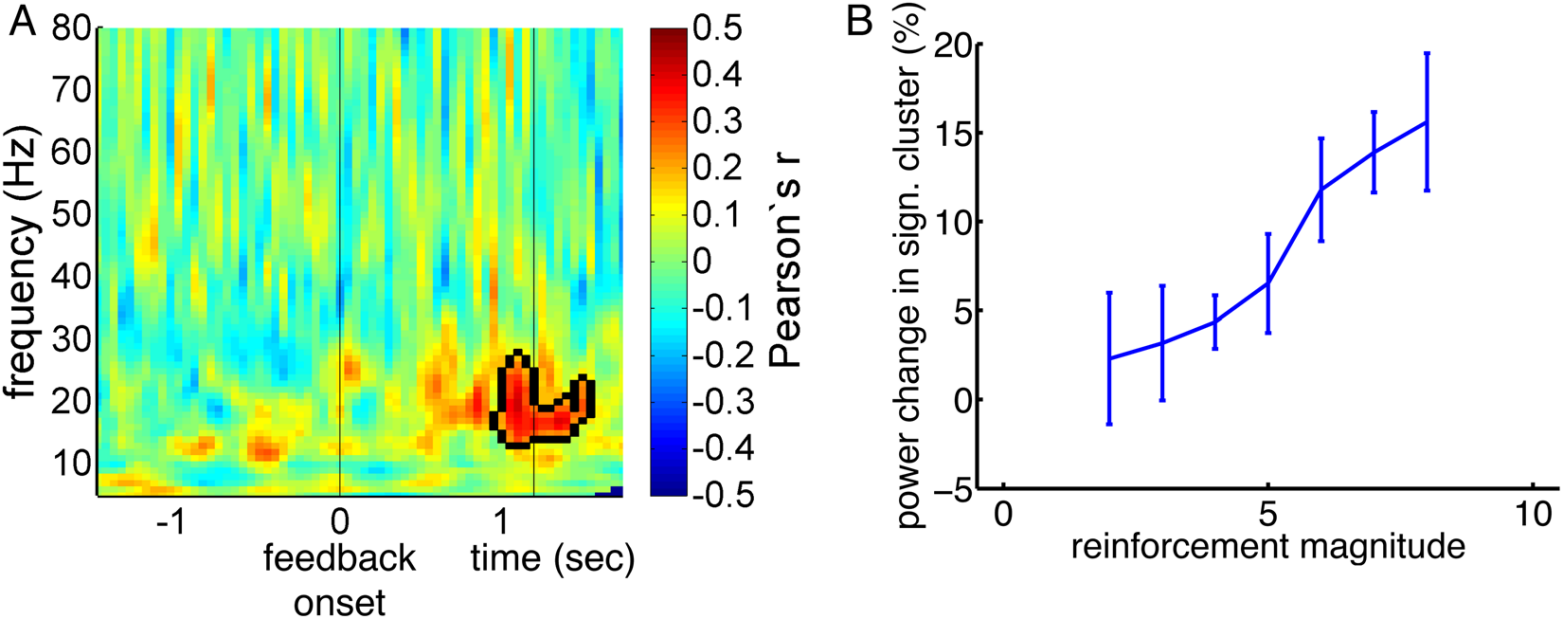
Reinforcement magnitudes modulate beta activity during feedback presentation. (**A**) Across time-frequency space, Pearson’s correlation coefficients between reinforcement magnitudes and task-related changes in LFP power are shown. Time point zero corresponds to feedback onset, time-point 1.2 to feedback offset. The frequency range spans 5 to 80 Hz. Correlations were tested for significance with a cluster-based approach described by Maris and Oostenveld^18^. Borders of a significant cluster are highlighted by a black line. (**B**) Average LFP power changes within the significant cluster of panel A relative to baseline are shown for different reinforcement magnitudes.

To investigate whether reinforcements modulate beta activity, we computed correlations between reinforcement magnitudes and baseline-corrected wavelet energy. For each reinforcement magnitude between the values of two and eight, we first performed a separate wavelet analysis; values below two and above eight (which were relatively rare due to Gaussian feedback probability curves) were included into the categories of two and eight, respectively. Across the resulting seven time-frequency plots, we correlated reinforcement magnitudes with LFP (wavelet) energy, separately for each patient. In a second step, we searched for significant clusters of correlations within time-frequency space across patients^18^. We observed a cluster of significant positive correlations between 500 and 1500 ms after feedback onset in the frequency range of 14 to 27 Hz (Fig. 4A), *p* = 0.049. The average correlation within this cluster (i.e., the mean of all individual correlation values within the cluster) was r = 0.30. Plotting average LFP power changes within this cluster separately for different reinforcement magnitudes, we observed increases in beta power relative to baseline in large-feedback trials, but no deviation from baseline in small-feedback trials (Fig. 4B).

Moreover, we analyzed LFP power changes across trials within blocks of constant response-outcome mappings. That is, we cut our overall trial series into several sub-series starting after each switch in stimulus-outcome mappings. Within each sub-series, we numbered each trial in ascending order and then binned together all trials with the same number across sub-series. For each trial number, we performed a separate wavelet analysis and then averaged LFP power changes across all time-frequency data points that fell into the significant cluster of Fig. 4A (Supplementary Fig. S3; please compare to Fig. 2A). No significant correlation was found between trial number and beta activity, r = 0.12, *p* = 0.11, suggesting that average beta activity does not change significantly across learning.

To rule out the possibility that our significant correlations between reinforcement magnitudes and LFP oscillations were confounded by movement parameters, we correlated our LFPs with response latencies and durations in equivalent ways. Neither response durations, *p* = 0.54 (Supplementary Fig. S4A), nor response latencies, *p* = 0.26 (Supplementary Fig. S4B) correlated significantly with baseline-corrected LFP oscillations, excluding these parameters as potential confounds.

**Figure 5:**
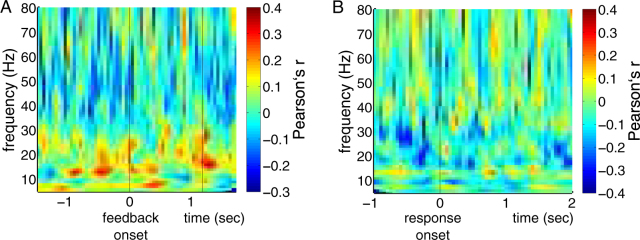
Reinforcement prediction errors do not modulate STN oscillations. (**A**) Pearson’s correlation coefficients between reinforcement prediction errors and feedback-locked changes in LFP power are shown across time-frequency space. Time point zero corresponds to feedback onset, time-point 1.2 to feedback offset. (**B**) Correlations between reinforcement prediction errors and *response-locked* changes in LFP power are shown across time-frequency space. The frequency range spans 5 to 80 Hz. Correlations were tested for significance with a cluster-based approach described by Maris and Oostenveld^18^.

To investigate whether reinforcement prediction errors (which well reflect phasic dopamine signals) modulate STN oscillations, we correlated these prediction errors with baseline-corrected wavelet energy. A reinforcement learning model as detailed in section 4.5 was fitted to patients’ individual behavioral performance, resulting in a separate estimation of the reinforcement prediction error for each trial and patient. For each patient, trials were then sorted into one of seven bins according to the magnitudes of the prediction errors. For each of these bins, a separate wavelet analysis was performed. Across the resulting seven power plots, reinforcement prediction errors were correlated with LFP energy, separately for each time-frequency bin (resolution of 1 Hz and 50 ms). Afterwards, we searched for significant clusters of correlations across patients (second-level analysis)^18^. In the resulting correlation plot (Fig. 5A), we did not observe any significant cluster of correlations in time-frequency space, *p* = 0.52, arguing against the assumption that reinforcement prediction errors, and therefore phasic changes in dopamine, modulate beta activity.

**Figure 6:**
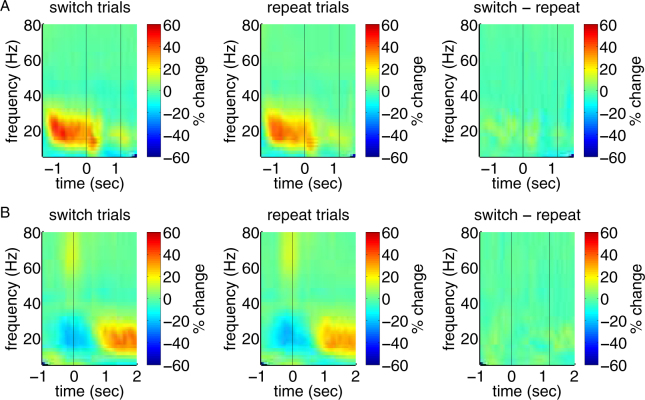
Feedback- and response-locked LFPs do not differ between switch and repeat trials. (**A**) For feedback-locked LFPs, average time-frequency maps are shown for all trials in which patients changed versus repeated their previous trials’ responses. Moreover, the difference map is shown. Time point zero corresponds to feedback onset, time point 1.2 to feedback offset. (**B**) For response-locked LFPs, average time-frequency maps are shown for all trials in which patients changed versus repeated their previous responses. Moreover, the difference map is depicted. Time point zero corresponds to response onset. Correlations were tested for significance with a cluster-based approach described by Maris and Oostenveld^18^.

To investigate whether STN oscillations were modulated by patients’ tendencies to maintain versus adapt their responses (status quo theory), we compared LFP oscillations of trials in which responses were switched to those in which responses were repeated (Fig. 6A). When looking for significant differences between these conditions^18^, we did not find any significant cluster, *p* = 0.09.

### Correlations of response-locked LFPs with behavioral parameters

**Figure 7:**
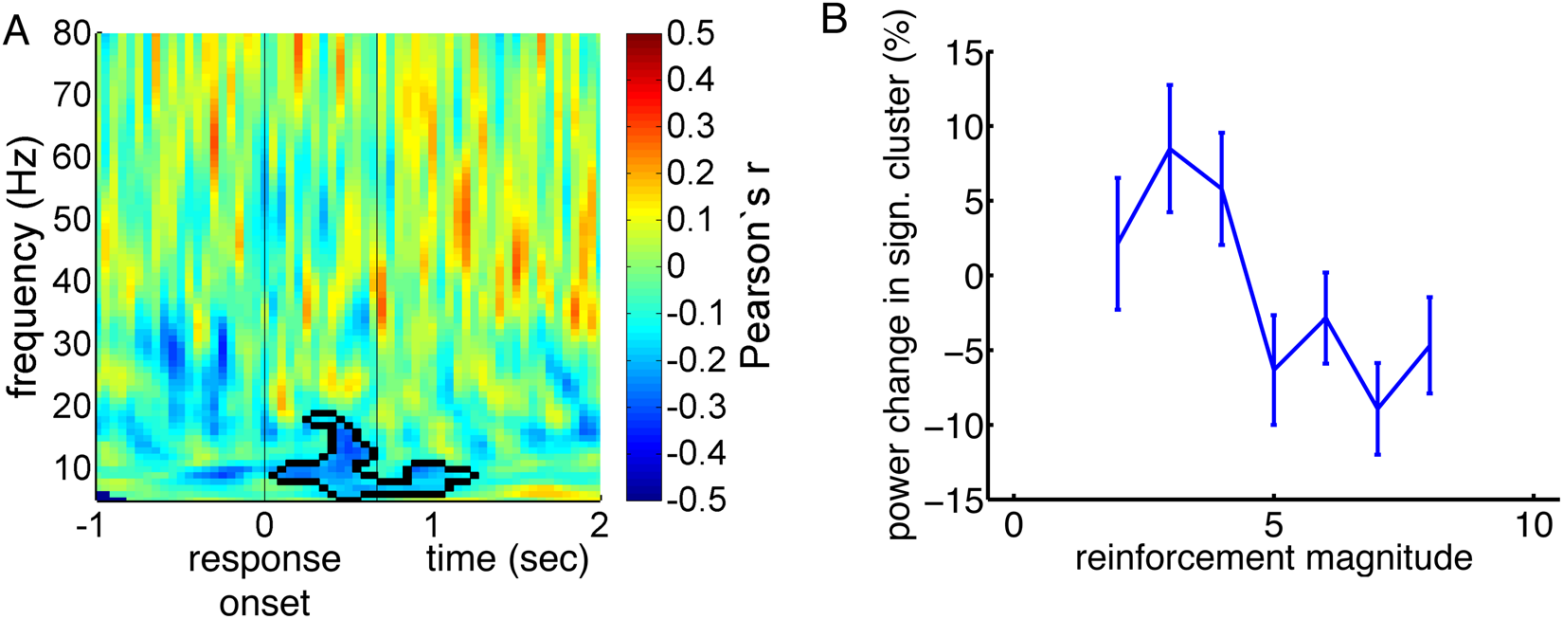
Reinforcement magnitudes of a given trial modulate alpha/low beta activity during the next trial’s joystick movement. (**A**) Across time-frequency space, Pearson’s correlation coefficients between reinforcement magnitudes and task-related changes in LFP power are shown. Time point zero corresponds to response onset; average response duration is depicted by a vertical line at 669 ms. The frequency range spans 5 to 80 Hz. Correlations were tested for significance with a cluster-based approach described by Maris and Oostenveld^18^. The borders of a significant cluster are highlighted by a black line. (**B**) Average LFP power changes within the significant cluster of panel A relative to baseline are shown for different reinforcement magnitudes.

Next, we investigated whether reinforcement magnitudes modulated STN oscillatory activity during subsequent joystick movements (Fig. 7A). We observed a significant negative correlation in the alpha/low beta spectrum in the frequency range of 6 to 18 Hz between response onset and approximately 1200 ms afterwards, *p* = 0.02. The larger the reinforcement obtained in a given trial, the lower the alpha/low beta activity during the following trial’s joystick movement. The average correlation within this cluster was r = −0.22. By plotting average LFP power changes within this significant cluster for different reinforcement magnitudes, we observed a decrease in alpha/low beta power for highly reinforced trials relative to baseline, and an increase for trials with small reinforcements (Fig. 7B). Computing separate time-frequency plots for the different reinforcement magnitudes (Supplementary Fig. S5), the significant correlation appeared to result from the peri-movement beta decrease stretching into lower frequencies between response onset and 1,200 ms afterwards for large, but not for small reinforcement magnitudes.

Again, we investigated whether these results could be explained by response latencies, durations or choices. For response durations, we observed a significant correlation with baseline-corrected LFP power between 500 and 1,200 ms after response onset in the frequency range of 11 to 27 Hz, *p* = 0.01 (Supplementary Fig. S6A). The average correlation within this cluster was r = −0.32. Though significant, however, this cluster does not overlap in time-frequency space with the cluster related to reinforcement magnitudes. Response durations, therefore, did not likely impact on these results. For response latencies, we indeed observed a significant correlation with beta oscillations in the time interval prior to 200 ms before response onset in the frequency range of 9 to 31 Hz, *p* < 0.001 (Supplementary Fig. S6B). The average correlation within this significant cluster was r = −0.28. Again, the cluster differed in time-frequency space from our cluster related to reinforcement magnitudes, arguing against response durations having impacted on these results.

Next, we investigated correlations between reinforcement prediction errors (which well reflect phasic dopamine levels) and subsequent response-locked LFP oscillations (Fig. 5B). These analyses did not produce a significant cluster in time-frequency space, *p* = 0.10.

To investigate whether LFP oscillations were modulated by patients’ tendencies to maintain versus adapt their response strategies (status quo theory), finally, we contrasted LFP power for trials in which patients switched versus repeated the previous trial’s response (Fig. 6B). We did not find a significant cluster of differences, *p* = 0.06.

## Discussion

We showed a task-related modulation of response- and feedback-locked STN oscillations by reinforcement magnitudes in human Parkinson’s disease patients during reinforcement learning. We did not, in contrast, find a modulation of these oscillations by reinforcement prediction errors (related to phasic dopamine signals) or by patients’ propensities to repeat versus adapt their responses based upon reinforcements (status quo theory).

### Effects of reinforcement magnitudes on LFP oscillations

During feedback presentation, the power of oscillations in the frequency range of 14 to 27 Hz was positively correlated with reinforcement magnitudes. These results were not due to confounding effects by response latencies or durations. During responding, moreover, the power of oscillations in the frequency range of 6 to 18 Hz (alpha and low beta spectrum) was negatively correlated with previous reinforcement magnitudes. Although for these response-locked results we observed significant correlations of LFP oscillations with response latencies and response durations, these did not overlap in time-frequency space with our significant clusters, arguing against confounding (or mediating) effects by these movement parameters. Still, however, our significant correlations might have been related to the adaptation of other types of response parameters that we did not record in our study. In fact, correlations between *response*-locked LFPs and previous reinforcement magnitudes might rather favor such an interpretation. Response parameters that might be of relevance here, but that we did not record, are the balance between response speed and accuracy^19,20^, motor effort^21^ and gripping force^22^ – where the time interval of our significant correlations particularly favors the latter.

A role of beta activity in feedback processing had been previously suggested based upon EEG data^6,23^. Large reinforcements were shown to go along with phasic increases in beta activity at frontocentral EEG electrodes in a gambling task, while large losses were accompanied by decreases in theta power at frontocentral sites^6^. These EEG effects agree with our intracranial results that large reinforcements cause phasic increases in beta activity in a reinforcement learning paradigm, while small reinforcements do not cause deviations of beta from baseline. Our findings extend these previous results by showing a reinforcement-based modulation of beta activity subcortically, i.e. in the STN, and by showing that reinforcements modulate alpha and low beta activity during subsequent responses.

In a previous LFP study in human Parkinson’s disease patients, reinforcements modulated oscillations in the frequency range below 10 Hz in the STN, thus not including beta activity^7^. In this study, an effort-based decision task was used, rather than a reinforcement learning paradigm as in our study. A role of STN beta activity in reinforcement learning has not been shown previous to our results. However, reinforcements have been repeatedly observed to modulate gamma oscillations in the ventral striatum of rats^8–11^. Because of a different target nucleus, however, these results cannot be easily compared to our findings.

### Potential effects of dopamine

Although we did not directly measure dopamine in this study due to obvious technical difficulties, phasic dopamine levels are well reflected by reinforcement prediction errors^4^. Jenkinson and Brown^5^ had hypothesized that the effects of phasic dopamine on beta activity would most likely be equivalent to known effects of tonic dopamine^3,24–26^. This assumption implies that large reinforcements which phasically increase dopamine emission in the basal ganglia for several seconds^27–29^ should decrease beta activity, while small reinforcements which phasically decrease dopamine should increase beta activity^5^. These predictions, however, do not match with our results which instead suggest the opposite relationship: large reinforcements increased beta activity, while small reinforcements did not cause deviations of beta from baseline. Either, our results therefore argue for opposite effects of tonic and phasic dopamine levels on beta activity or they are unrelated to dopamine.

### Maintenance of the status quo

Increases in beta activity have been implicated with neuronal commands to maintain the status quo, i.e. the current sensorimotor or cognitive state of the brain^17^. This theory is based on evidence that phasic decreases in STN beta activity occur during movements (Fig. 3)^12^, while phasic *increases* in beta activity can be observed directly *after* movement execution (see also Fig. 3) and under circumstances where intended movements are withheld^12,30^. Applied to the context of reinforcement learning, this theory would predict that beta activity is higher in trials in which patients repeat previous responses (i.e., maintain the status quo) than in trials in which responses are adapted. We could not confirm this prediction based on our results. Overall therefore, our results do not provide support for the status quo theory.

### Limitations

Our LFPs were recorded from Parkinson’s disease patients who are known to suffer not only from motor, but also from cognitive and motivational dysfunctions [e.g.^31–34^]). Of particular interest to the interpretation of our results, they are known to be impaired in the evaluation of feedback^35^, learning more easily from negative, but less easily from positive outcomes than healthy control subjects^36^. Therefore, it remains speculative whether our findings generalize to healthy participants from whom such intracranial LFPs cannot be recorded. In favor of generalizability, however, we want to point out that our patients readily learned the paradigm without any observable impairments and that our results are in line with previous EEG findings from healthy participants as discussed above.

Similarly, the effects of dopamine medication on phasic beta activity remain unknown. We cannot exclude the possibility that our results would have turned out different with unmedicated patients. We chose to record our patients on medication both, because our paradigm was easier to grasp and perform for patients in that state and because tonic beta activity in this state is thought to resemble healthy subjects’ beta activity more closely^24^. In comparison to unmedicated patients, however, tonic beta activity is suppressed^1–3^. It would thus be important to study reinforcement-related beta band modulation in unmedicated patients in the future.

Moreover, the reinforcement learning model used in our analyses is not specifically tuned to the reinforcement characteristics of our task, i.e., the fact that reinforcement probability curves have fixed means of 2.5, 5.0 and 7.5 and standard deviations of 1.0. Our patients probably found out these characteristics as they became familiar with the task and made use of this knowledge when adapting to contingency changes. The model, in contrast, is incapable of learning (and remembering) such task characteristics. Also, the model has fixed learning rates across all trials (one learning rate for trials with positive reinforcement prediction errors and another for trials with negative prediction errors). Empirically, however, there has been evidence arguing for a dynamic adaptation of learning rates according to the volatility of reinforcement contingencies in humans^37^.

Finally, all reinforcement learning paradigms are inherently non-experimental in nature (i.e., they do not allow for an active manipulation of variables independent of subjects’ behavior). As a consequence, all observed correlations between LFP oscillations and behavioral parameters could have in principle been confounded by other variables that covary with these parameters. In our analyses, we tested whether response latencies and durations had confounded our results, but did not find any such evidence. These analyses do not exclude other potential confounds.

### Conclusions

Our results suggest that in Parkinson’s disease patients, STN alpha and beta oscillations during reinforcement learning reflect these patients’ evaluation of reinforcement magnitudes and their subsequent adaptation of response parameters based on this evaluation. We did not find evidence for a modulation of beta activity by reinforcement prediction errors or by patients’ tendencies to repeat versus adapt their response choices. We therefore conclude that alpha and beta activity in reinforcement learning truly reflects patients’ processing of reinforcement magnitudes, but does not reflect the effects of phasic dopamine signals or patients’ tendencies to maintain the status quo.

## Material and Methods

The experimental protocol was approved by the local ethics committee (Charité – University Hospital Berlin). The study was carried out in accordance with all relevant guidelines and regulations. Informed consent was obtained from all patients.

### Patients and surgery

19 patients suffering from idiopathic Parkinson’s disease were included in our analyses (mean age: 59.7 years, SD: 9.2 years; mean onset age: 48.7 years, SD: 8.8 years). Detailed patient characteristics are given in Table 1. Two additional patients had quit the investigation due to difficulties in concentrating after performing only a few trials; these were excluded from all analyses. All patients had undergone surgery for implantation of DBS electrodes into the STN between one and four days before participating in our study. The pulse generator, however, had not yet been implanted and electrode leads were still externalized, giving us the opportunity to record LFPs. Twelve patients were operated at Charité – University Medicine Berlin, seven at Medical University Hanover. Patients were implanted with macro-electrodes model 3389 (Medtronic Neurological Division, MN, USA). This electrode contains four cylindrically shaped platinum-iridium contacts (diameter: 1.27 mm, length: 1.5 mm) with a distance between contacts of 0.5 mm. 18 patients were implanted bilaterally, a single patient unilaterally in the left hemisphere. Electrode positions of 17 patients were localized post-operatively using LEAD-DBS software (www.lead-dbs.org)^37^.

**Table 1:** Overview of clinical data.

Patient no (sex)	Operating center	Age	Onset age	Preoperative medication	Postoperative medication	Preoperative UPDRS III motor score OFF med.	Preoperative UPDRS III motor score ON med.	Contact pairs excluded from LFP analysis	Stimulation settings
1 (m)	H	66	50	l-dopa 600 mg/d pramipexole 1.75 mg/d rotigotine 4 mg/d bornaprine 12 mg/d opipramol 100 mg/d rasagiline	rotigotine 4 mg/d bornaprine 6 mg/d rasagiline	27	8	none	1–1.9 V; 11–2.2 V; 130 Hz, 60 µS
2 (m)	H	69	58	l-dopa 550 mg/d pramipexole 2.1 mg/d amantadine 200 mg/d domperidone 20 mg/d	pramipexole ret. 2.1 mg/d domperidone 20 mg/d	35	25	none	1–1.6 V; 9–3.0 V; 180 Hz, 60 µS
3 (m)	H	66	59	l-dopa 850 mg/d pramipexole 2.1 mg/d clozapine 25 mg/d	l-dopa 625 mg/d	39	30	none	2–3.6 V; 9–3.6 V; 130 Hz, 60 µS
4 (m)	H	37	28	l-dopa 800 mg/d apomorphine 124 mg/d rasagiline	l-dopa 500 mg/d rasagiline	37	44	none	3–4.3 V; 11–2.3 V; 130 Hz; 60 µS
5 (m)	H	58	51	pramipexole ret 3.15 mg/d	pramipexole 1.575 mg/d	24	8	none	2–2.3 V; 10-/11–2.6 V; 130 Hz; 60 µS
6 (m)	B	69	44	l-dopa 150 mg/d pramipexole ret 4.2 mg/d amantadine 400 mg/d	l-dopa 375 mg/d amantadine 400 mg/d	26	13	none	2+/3–3.7 V; 10+/11–3.0 V; 130 Hz; 60 µS
7 (f)	B	47	40	l-dopa 600 mg/d ropinirole 16 mg/d piribedil 150 mg/d tolcapone 300 mg/d rasagiline	n.d.	n.d.	18	L12	2–2.0 V; 10–1.6 V; 130 Hz, 60 µS
8 (m)	B	50	43	l-dopa 400 mg/d pramipexole ret 3.15 mg/d rasagiline	pramipexole 2.1 mg/d rasagiline	27	7	none	1–2.1 V; 9–2.5 V; 140 Hz; 60 µS
9 (f)	B	58	50	l-dopa 400 mg/d pramipexole 2.8 mg/d	pramipexole ret 0.52 mg/d	34	24	none	2–1.0 V; 10–2.5 V; 130 Hz; 60 µS
10 (m)	B	54	47	l-dopa 1200 mg/d	no l-dopa	14	3	L01, R01, R12, R23	2–4.5 V; 130 Hz; 60 µS
11 (m)	B	56	38	l-dopa 400 mg/d amantadine 450 mg/d rasagiline	l-dopa 300 mg/d amantadine 450 mg/d rasagiline	41	20	none	1–3.2 V; 9–2.0 V; 130 Hz; 60 µS
12 (m)	H	53	44	l-dopa 500 mg	l-dopa 187.5 mg/d	21	13	L23	2–0.5 V; 10–1.1 V; 130 Hz; 60 µS
13 (f)	H	52	41	l-dopa 900 mg/d ropinirole 16 mg/d	l-dopa 400 mg/d	26	18	none	2–1.2 V; 10–2.2 V; 130 Hz, 60 µS
14 (f)	B	66	55	l-dopa 425 mg/d pramipexole 4.2 mg/d rasagiline	pramipexole ret 3.15 mg/d	34	11	none	2–1.4 V; 10–1.6 V; 130 Hz; 60 µS
15 (m)	B	62	53	l-dopa 900 mg/d tolcapone 600 mg/d rotigotine 4 mg/d	l-dopa 500 mg/d	31	14	none	0–2.0 V; 8–2.0 V; 130 Hz, 60 µS
16 (m)	B	72	57	l-dopa 1050 mg/d piribedil 150 mg/d	l-dopa 500 mg/d piribedil 50 mg/d	59	43	R12, R23	2–2.6 V; 10–2.2 V; 130 Hz; 60 µS
17 (m)	B	68	n.d.	l-Dopa 700 mg/d ropinerole 16 mg/d amantadine 200 mg/d rasagiline1mg/d	l-dopa 500 mg/d amantadine 200 mg/d rasagiline1mg/d	37	27	none	1–2.8 V; 9–2.0 V; 130 Hz, 60 µS
18 (m)	B	64	55	l-dopa 400 mg/d pramipexole ret 3.15 mg/d amantadine 300 mg/d rasagiline	l-dopa 300 mg/d pramipexole ret. 0.52 amantadine 100 mg/d rasagiline	43	20	none	1–2.7 V; 9–3.4 V; 110 Hz; 60 µS
19 (m)	B	67	63	l-Dopa 1300 mg/d rotigotine 6 mg/d	l-dopa 800 mg/d rotigotine 6 mg/d	26	17	none	1–2.7 V; 9–2.2 V; 130 Hz; 60 µS

### Electrode localization and mapping of electrophysiological values on MNI space

**Figure 8:**
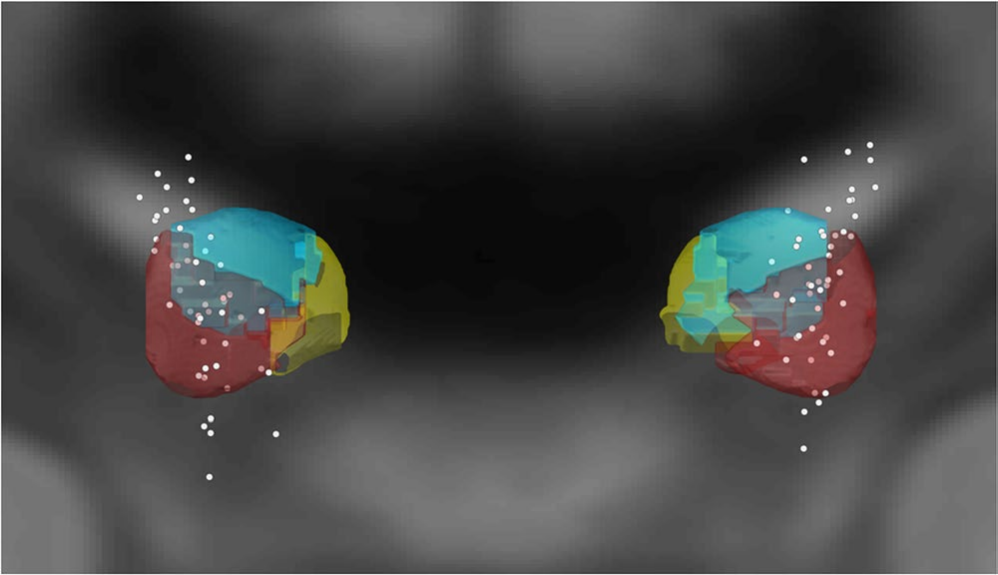
Spatially reconstructed positions of all contact pairs from which STN LFPs were bipolarly recorded. For orientation, motor, associative and limbic parts of the STN, as defined in the atlas by Accola *et al*.^40^ are shown in red, cyan and yellow, respectively.

Electrode leads were localized using Lead-DBS software^38^. Postoperative stereotactic CT images (Hanover patients) or MRI images (Berlin patients) were co-registered to preoperative MRI images using SPM12 (MR modality) and BRAINSFit software (CT modality) with an affine transform. Images were then nonlinearly warped into standard stereotactic (MNI; ICBM 2009 non-linear) space using a fast diffeomorphic image registration algorithm (DARTEL)^39^. Finally, electrode trajectories were automatically pre-localized and results were manually refined in MNI space using Lead-DBS. All electrodes were confirmed to correctly lie within the STN. Figure 8 depicts the spatial locations of all individual channels from which our data were recorded. Each recording channel was localized at the center of the two contacts from which bipolar recordings were taken. For comparison, motor, associative and limbic STN sub-regions are shown as well, as based on an atlas by Accolla^40^. Recordings channels can be seen to cluster around the STN’s motor-associative border region.

### Recording setup and procedure

Patients were seated in a comfortable chair in a well-lit recording room. A joystick was placed on a desk in front of participants such that they could comfortably move it with the hand of their choice. 13 patients used their right hand, four patients their left hand and two patients alternated between left and right hands. Stimuli were presented on a laptop computer screen that was placed behind the joystick, approximately 100 cm away from patients’ eyes. Patients gave informed consent prior to participation.

### Behavioral paradigm

Patients performed a computer-based reinforcement learning game that involved frequent reinforcement reversals. They were asked to maximize reinforcements by choosing appropriate responses (Fig. 1A). In each trial, a red square was presented first. The square remained on the screen until patients had not moved the joystick for a continuous interval of 1,500 ms, allowing us to obtain an uninterrupted 1,500 ms interval without motor artefacts. This interval served as a baseline period for LFP analyses. As soon as the 1,500 ms were complete, the fixation square changed its color to green, prompting patients to move the joystick into one of three directions (left, right or front). Afterwards, the red square appeared again and remained on the screen until the joystick had not been moved from its center position for another 1,500 ms. In other words, the red square was presented for at least 1,500 ms, but if patients had moved the joystick within this interval (against instructions), the interval was extended until 1,500 ms without motor artefacts had been obtained. This ensured that the subsequent interval of feedback presentation was unaffected by any movement-related or post-movement changes in beta activity. Patients were then presented with a number between 0 and 10 (reinforcement magnitude) for 1,200 ms, followed by an inter-trial-interval (ITI) with an average duration of 1,500 ms (SD: 250 ms, min: 1,000 ms).

Reinforcement magnitudes presented to patients were determined via Gaussian probability distributions, where each movement direction was associated to one particular distribution (Fig. 1B). Based on the chosen direction, a reinforcement magnitude was randomly drawn from the corresponding distribution in each trial. The three distributions differed in means (2.5, 5 and 7.5), but had equal standard deviations of 1. Every 20 trials on average (SD: 3), probability distributions were randomly interchanged between movement directions without notice to patients.

### Behavioral analyses and reinforcement-learning model

Patients’ response latencies and durations were recorded and analyzed. Response latencies were defined as the interval between the ‘Go’ signal prompting patients to perform their response (green fixation square) and response onset (joystick deflection to at least 80% of its maximal value). Response durations were defined as the time interval between response onset and the joystick’s return to its center position.

To estimate reinforcement prediction errors from patients’ behavior, we fitted a canonical reinforcement-learning model to each patient’s individual response timeline [see^41–44^]. The model was based on the following equations:

1 $$er{r}_{t}=re{w}_{t}-pre{d}_{r,t}$$

with:

2 $$\begin{array}{c}pre{d}_{r,t+1}=pre{d}_{r,t}+{\partial }^{{\rm{pos}}}\,\ast \,er{r}_{t},\,{\rm{if}}\,{{\rm{err}}}_{{\rm{t}}} > 0\\ pre{d}_{r,t+1}=pre{d}_{r,t}+{\partial }^{{\rm{neg}}}\,\ast \,er{r}_{t}\,else.\end{array}$$

In these equations, *err*_*t*_ signifies the prediction error at time *t*, *rew*_*t*_ the actual reward at time *t*, *pred*_*r,t*_ denotes the reinforcement prediction for selecting the selected response *r* at time *t* and ∂^pos^ and ∂^neg^ are the learning rates for trials with positive and negative reinforcement prediction errors, respectively. Reinforcement predictions for unselected responses • were changed in opposite ways via:

3 $$\begin{array}{c}pre{d}_{\bullet ,t+1}=pre{d}_{\bullet ,t}-0.5\,\ast \,{\partial }^{{\rm{pos}}}\,\ast \,er{r}_{t},\,{\rm{if}}\,{{\rm{err}}}_{{\rm{t}}} > 0\\ pre{d}_{\bullet ,t+1}=pre{d}_{\bullet ,t}-0.5\,\ast \,{\partial }^{{\rm{neg}}}\,\ast \,er{r}_{t}\,{else}{\rm{.}}\end{array}$$

In each new trial of the paradigm, reinforcement predictions and reinforcement prediction errors were updated according to patients’ selected responses and received reinforcements. The parameters ∂^pos^ and ∂^neg^ were fitted to patients’ performance across trials such that the model correctly predicted patients’ selected responses in the largest possible number of trials (where the model was assumed to predict the response which was associated with the largest reward prediction value of all response options at the relevant time point). For both ∂^pos^ and ∂^neg^, a full search in the parameter space between 0.01 and 1.00 with a step size of 0.01 was performed. If there was more than one combination of ∂^pos^ and ∂^neg^ that produced equally good fits, that combination was selected whose distribution of reinforcement prediction values differed the least from a uniform distribution of [2.5, 7.5].

Prediction errors were then correlated with STN activity as detailed in the following sub-section.

### Recording and analyses of LFP data

LFPs were recorded bipolarly (online) from all adjacent contact pairs of each electrode. No offline re-referencing was done. Sampling frequency was 5,000 Hz. LFPs were amplified with a factor of 50,000 and bandpass filtered between 0.5 and 1,000 Hz using a Digitimer D360 (Digitimer Ltd., Welwyn Garden City, Hertfortshire, UK). All recordings were initially saved in Spike 2 (Cambridge Electronic Design). Off-line, they were filtered with a 50 Hz notch filter to remove powerline noise and then exported to Matlab® (The Mathworks) for all analyses.

LFP data were pre-processed for artefacts in a two-step procedure. First, channels in which voltages repeatedly reached the recording boundaries of ±100 µV were completely excluded from all analyses based upon visual inspection. Secondly, we excluded all trials in which the voltage in one of the remaining channels exceeded ±90 µV. Artefact trials were excluded from analyses of both LFP and behavioral data. LFP data were then cut into trial-related epochs relative to response and feedback onsets.

LFP data were analyzed with regard to task-related changes in spectral power. Using the FieldTrip toolbox^45^ in Matlab® (The Mathworks), we computed wavelet energy using Morlet wavelets with seven wavelet cycles. The wavelets’ length was chosen as three times the standard deviation of the implicit Gaussian kernel. Frequencies were sampled between 5 and 80 Hz with a step-size of 1 Hz.

To compute grand-average time-frequency plots (as presented in Fig. 3), wavelet energy was computed separately for the response-locked time window of interest, the feedback-locked time window of interest and for the baseline interval, each at a step-size of 50 ms and separately for each contact pair. Task-related changes in wavelet energy within the time windows of interest were then computed relative to the average baseline energy (i.e., the mean energy across all time-points of the baseline interval, separately for each frequency bin). Next, task-related changes in energy were averaged across contact pairs within patients. Clusters of power changes were then tested for significance across patients with the non-parametric, permutation-based technique described by Maris and Oostenveld^18^. Grand-average time-frequency plots were computed by averaging individual time-frequency plots across patients.

Correlations between task-related changes in wavelet energy and behavioral parameters (e.g., reinforcement magnitudes, reinforcement prediction errors, response latencies or response durations) were computed with the following procedure. In a first step, separate wavelet analyses were performed for each patient, recording channel and behavioral parameter value of interest, as outlined in the preceding paragraph. For reinforcement magnitudes, parameter values of interest were [<=2, 3, 4, 5, 6, 7, > = 8], while reinforcement prediction errors, response latencies and response durations were binned into 7 bins of increasing parameter values (the lowest 14% of values went into the first bin, the next-lowest 14% into the second bin, etc.). For each combination of patient, recording channel and parameter value of interest, this resulted in a separate time-frequency matrix of changes in LFP power relative to the baseline period. After averaging across recording channels, behavioral parameter values (or, for binned data, average values of the different bins) were correlated with baseline-corrected LFP power separately for each time-frequency bin and for each patient (Pearson coefficient). Finally, the resulting time-frequency specific correlation values were tested for significance across patients (second-level analysis) with the non-parametric, permutation-based technique described by Maris and Oostenveld^18^.

### Statistics

For inference testing of time-frequency data, we used the cluster-based permutation statistics developed by Maris and Oostenveld^18^. The approach makes no assumptions on the distribution of the underlying data and offers full control of the family-wise error rate (FWER) at the relevant alpha level of 0.05. In brief, the approach involves the following steps. First, we processed the original data by computing a separate *t*-test (18 degrees of freedom) for each value in time-frequency space across patients. As outlined in the preceding sub-section, dependent values within the time-frequency space were either LFP power (see Fig. 3) or correlations between LFP power and behavioral parameters (see Figs 4, 5 and 7). Afterwards, all *t*-values above a threshold of *t* = 2.10, corresponding to a probability of 0.05 with 18 degrees of freedom, were identified. Please note that this probability is not related to the critical alpha level of the hypothesis test (also set to 0.05 in our analyses), but that it defines the sensitivity of the cluster threshold, i.e., the threshold that defines cluster boundaries for subsequent cluster analyses. For all clusters of neighboring above-threshold *t*-values, subsequently, the *t*-values within the respective cluster were summed up and this sum served as a test statistic for that cluster in subsequent analyses.

Now, the original time-frequency data were permuted 20,000 times to establish a distribution of data that the original data’s test statistic could be compared against. For each of the 20,000 permutations, each patient’s dependent values in time-frequency space were, randomly, either left unchanged or multiplied by −1 (uniformly across all dependent values of that patient). Afterwards, the across-patient *t* statistic was computed again, exactly as for the original data. For each permutation, only the most powerful cluster, i.e., the largest sum of neighboring t values was identified and saved, resulting in a distribution of 20,000 values. For each cluster of the original data set, finally, the rank of its sum of *t*-values within the distribution of summed *t*-values from the permuted data sets was established. This rank defined the *p* value for that cluster (see^18^).

For inference tests of all non-time-frequency data (i.e. all data that comprised one single dependent value per patient), we used a sign permutation test [see^46^]: to establish a *p* value, we computed the mean dependent value of the original data across patients and ranked it within the distribution of mean dependent values derived from a large number of permuted data sets. For each permutation, each of the 18 dependent values was either left unchanged or multiplied by −1. We evaluated all 524,288 possible permutations, since this procedure is not overly computationally intensive.

## Electronic supplementary material

Supplementary Figures
