# Supplementary material for: Reinforcement magnitudes modulate subthalamic beta band activity in patients with Parkinson’s disease
Source: Sci Rep. 2018 Jun 5;8:8621. doi: 10.1038/s41598-018-26887-3 (PMC5988736; doi:10.1038/s41598-018-26887-3)
Supplement: Supplementary file 1 — Supplementary Figures [file 41598_2018_26887_MOESM1_ESM.pdf]

Reinforcement magnitudes modulate subthalamic beta band activity in patients with Parkinson's disease

Henning Schroll, Andreas Horn, Joachim Runge, Axel Lipp, Gerd-Helge Schneider, Joachim K. Krauss, Fred H Hamker*, Andrea A Kühn*   *Equal contributions

Supplementary Material

Supplementary Figure S1

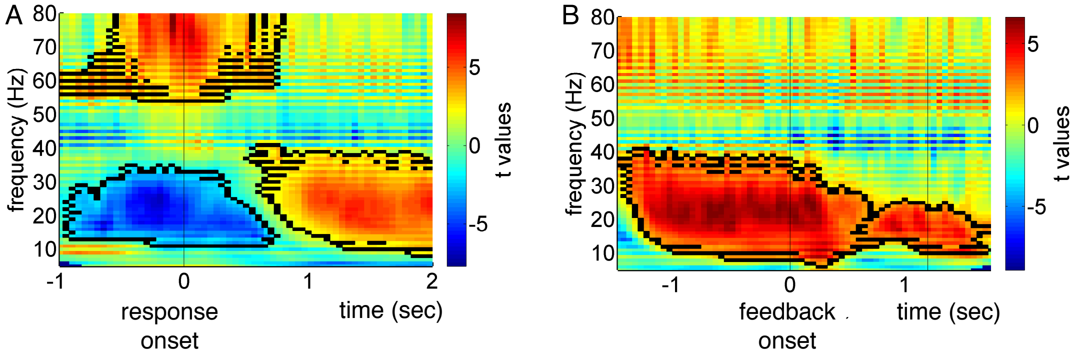

Supplementary Figure S1. Time-frequency maps of *t* values computed across patients for both response- and feedback-locked grand average LFP power changes. For each time-frequency bin, a separate dependent samples t test was computed across patients. Based on these *t* values, cluster-based inference tests were computed as described by [18]. A) Response-locked results. B) Feedback-locked results. Black lines highlight the outlines of significant clusters.

Supplementary Figure S2

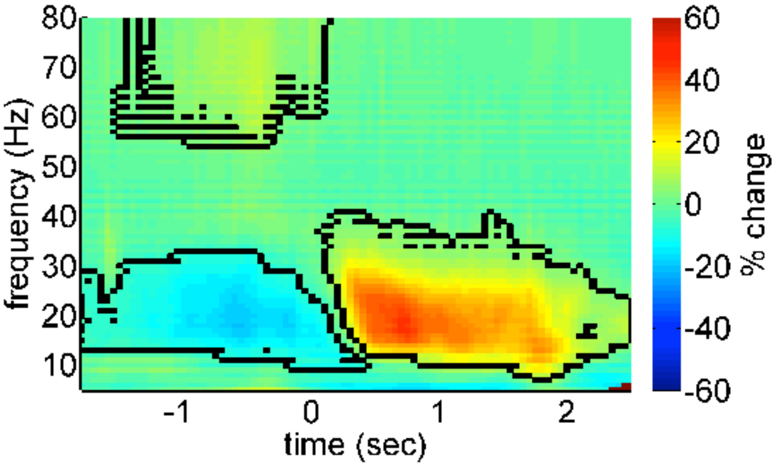

Supplementary Figure S2. Grand-average time-frequency plot of baseline-corrected oscillatory power locked to response termination (time point zero). Black lines show the borders of significant clusters as determined with the method by Maris and Oostenveld [18].

Supplementary Figure S3

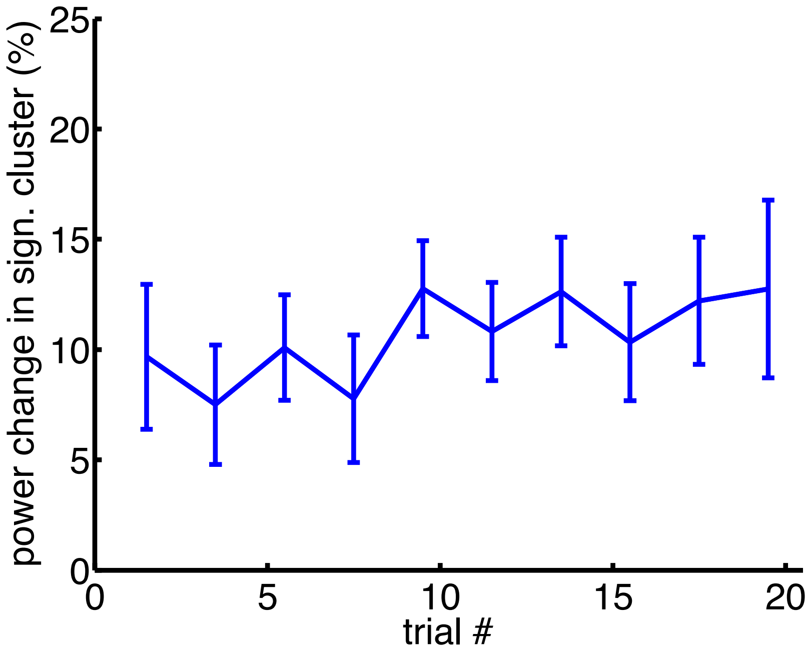

Supplementary Figure S3. Averages and standard deviations (across patients) of the power changes within the significant cluster of Figure 4 are shown across trials of constant response-outcome mappings. As detailed in the main text, there was no significant correlation.

Supplementary Figure S4

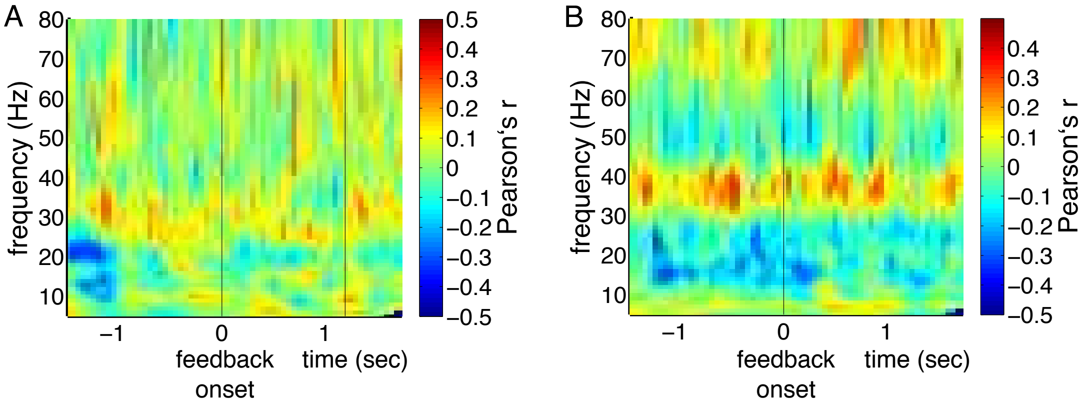

Supplementary Figure S4. Neither response durations nor response latencies correlate significantly with beta activity during feedback presentation. A) Across time-frequency space, Pearson's correlation coefficients between response durations and task-related changes in LFP power are shown. B) Across time-frequency space, Pearson's correlation coefficients between response latencies and task-related changes in LFP power are shown. Time point zero corresponds to feedback onset, time-point 1.2 to feedback offset. The frequency range spans 5 to 80 Hz. Correlations were tested for significance with a cluster-based approach described by Maris and Oostenveld [18]. There were no significant clusters.

Supplementary Figure S5

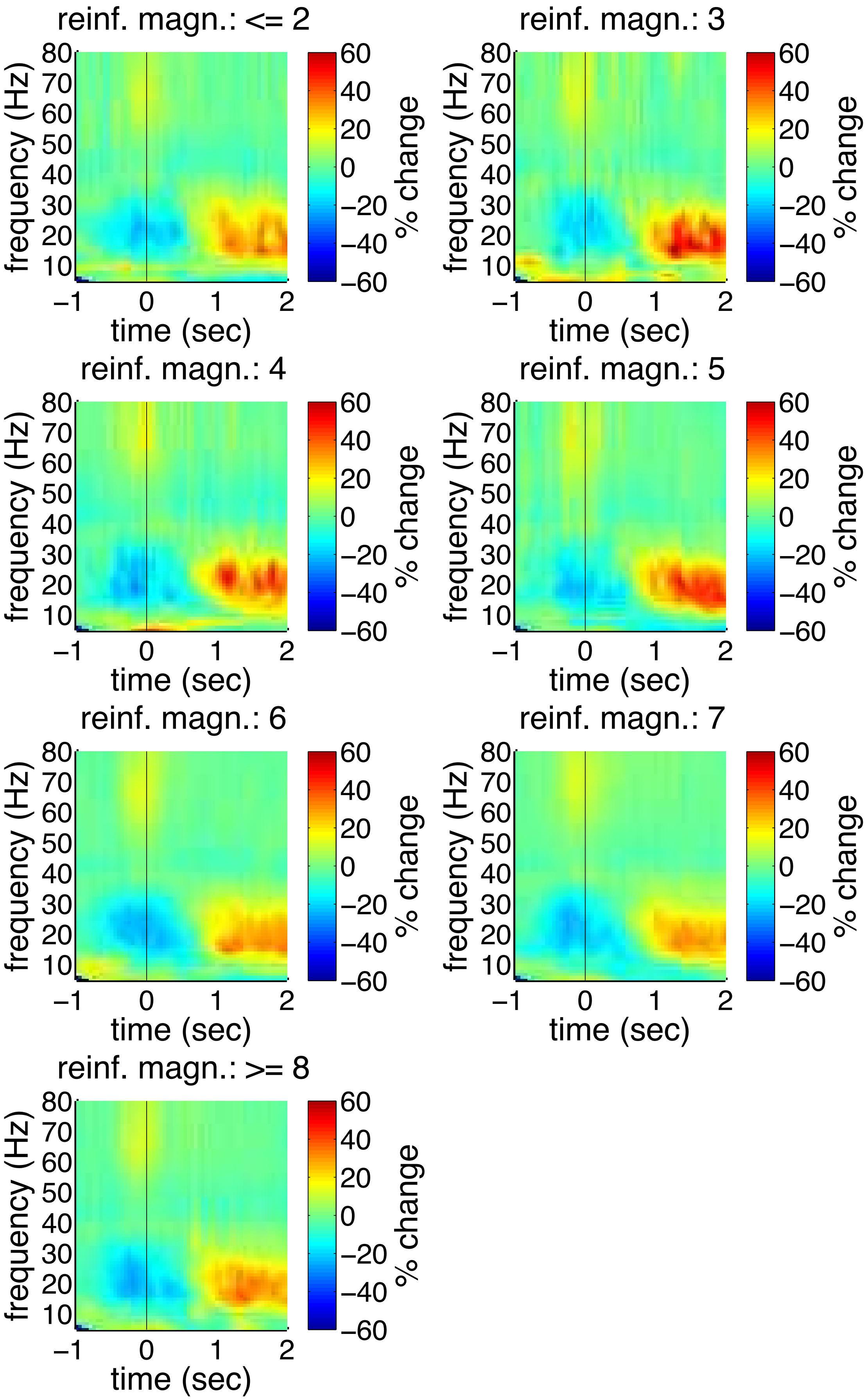

Supplementary Figure S5. Time-frequency plots show response-locked changes in oscillatory power relative to the baseline interval separately for different reinforcement magnitudes. Time point zero corresponds to response onset.

Supplementary Figure S6

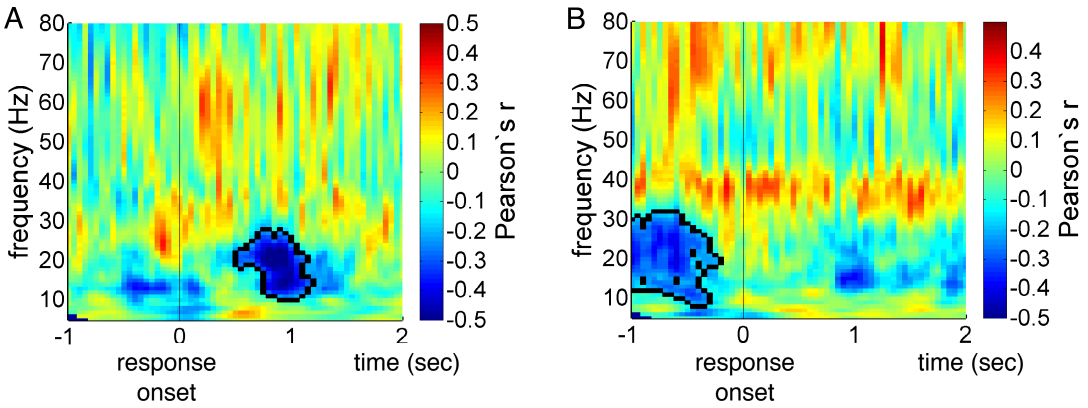

Supplementary Figure S6. Response durations and latencies correlate significantly with beta activity in response-locked LFP data. A) Across time-frequency space, Pearson's correlation coefficients between response durations and task-related changes in LFP power are shown. B) Across time-frequency space, Pearson's correlation coefficients between response latencies and task-related changes in LFP power are shown. Time point zero corresponds to response onset. The frequency range spans 5 to 80 Hz. Correlations were tested for significance with a cluster-based approach described by Maris and Oostenveld [18]. The borders of significant clusters are highlighted by black lines.
